# Allergenic Proteins in Enology: A Review on Technological Applications and Safety Aspects

**DOI:** 10.3390/molecules200713144

**Published:** 2015-07-21

**Authors:** Elena Peñas, Chiara di Lorenzo, Francesca Uberti, Patrizia Restani

**Affiliations:** 1 Institute of Food Science, Nutrition and Technology (ICTAN-CSIC), Juan de la Cierva 3, Madrid 28006, Spain; E-Mail: elenape@ictan.csic.es; 2 Dipartimento di Scienze Farmacologiche e Biomolecolari, Università degli Studi di Milano, via Balzzaretti 9, Milano 20133, Italy; E-Mails: chiara.dilorenzo@unimi.it (C.L.); uberti_francesca@libero.it (F.U.)

**Keywords:** winemaking, food allergens, allergenic residues, fining agents, egg proteins, milk proteins, isinglass

## Abstract

Proteinaceous products are widely used as fining agents during winemaking to remove unwanted insoluble particles and undissolved microscopic particles (colloidal material) from the must or wine to improve stability. Some of them (egg white, caseinates, and fish gelatine) have allergenic potential and the presence of their residues in the final product could represent a risk for allergic individuals. Moreover, lysozyme (an egg allergen) is included among wine additives to control the fermentation processes and avoid spoiling during winemaking. The aim of this paper is to review the experimental/clinical data on the use of allergenic products in enology and the measurement of relative risk for sensitized subjects. In addition, methods developed specifically for the quantification of allergenic residues in must and wine are described.

## 1. Introduction

Wine is a beverage resulting from the fermentation of grape must with appropriate processing and additives. The diversity and quality of wine result from the grape variety, soil composition, location, climate and the enological processes used. During vinification, grapes, must and wine are susceptible to various defects that significantly influence consumer acceptability. Consumer perception is usually related to the product appearance, flavor, color and composition, including the content of alcohol, acids, *etc.* The use of several products is permitted during winemaking. Some of them are additives and are still present in bottled wine; others are normally removed after treatment and do not leave any residue in the final beverage (the so-called processing aids).

Additives include substances that are added to wine to improve some characteristics, for example some (such as tartaric acid, malic acid, calcium carbonate, potassium bicarbonate) are used to increase or reduce the acidity or to improve wine structure and taste (such as tannins). Moreover additives (such as yeast, or yeast nutrients) ensure a rapid fermentation. Processing aids are normally used to ensure or enhance the stability of the final product; among them, fining agents are the most widely known.

Some additives and processing aids used in vinification are proteins, and some of them are provided by foods included amongst the most important allergens (such as milk proteins, egg white proteins, *etc.*). The use of allergenic proteins in enology raises the possibility of adverse reactions to treated wine in sensitized individuals.

## 2. Legislation

The European Union adopted Directive 2003/89/EC [1], last amended by Directive 2007/68/EC [2], which contains a list of allergenic substances (Annex III), that have to be declared on the label of foodstuffs. EC Directive 2005/26/EC [3] listed food ingredients that were provisionally excluded from the labeling requirements; this list, including all proteinaceous fining agents, was modified by Directive 2007/68/EC, which only allowed an exception for isinglass (fish gelatine) [2].

Finally, according to the EU Commission Implementing Regulation No. 579/2012 of 29 June 2012 (amending the Commission Regulation 607/2009), it was established that wines treated with allergenic additives or processing aids are subjected to specific labeling “*if their presence can be detected in the final product*” [4,5].

The new international rules make quantification of the residual allergenic agents absolutely necessary in order to determine the suitable labeling of wine bottles. As reported above, allergenic proteins used in enological processes are both additives and processing aids, although most of them are used in the second category as fining agents. Even if processing aids would not remain in the final product, their presence (also in trace amount) must be excluded in order to avoid any clinical reaction in allergic subjects.

## 3. Use of Allergenic Proteins as Processing Aids

Fining is one of the least expensive steps in wine production with a critical impact on wine quality. It aims to soften or reduce the wine astringency and/or bitterness; to clarify and remove proteins capable of haze formation; and/or to stabilize and reduce the color by the adsorption and precipitation of polymeric phenolic compounds and tannins [6]. Among the proteins used for fining, some are obtained from foods included in the list of main allergens in the EU Regulation: milk and egg proteins, animal gelatine, fish gelatine/isinglass and, more recently, proteins derived from plants such as wheat and white lupin [7,8].

### 3.1. Egg Proteins

#### 3.1.1. Enological Applications

Egg white proteins (commercially defined as “albumin” according to the OIV definition) is one of the most used fining agents in making red wines. It is positively charged and binds with negatively charged compounds such as tannins. The precipitate settles, and is removed mechanically from the wine by racking and/or filtration before bottling [9]; on the other hand, the low affinity of egg proteins for anthocyanin-tannin complexes has little influence on the red color. A secondary fining agent (such as the inorganic fining agent bentonite) can be used to remove any residual proteinaceous fining agent from the wine [10]. The quantity of dry egg white proteins usually added to red wine ranges between 3 and 15 g/hL (equivalent to 30–150 mg/L). Egg white contains several allergenic proteins as described in Table 1 [11,12].

**Table 1:** Main allergens of egg white (albumen).

Protein	International Allergen Code ^	Protein Content (% Total Protein)	Protein Content (g/100 g Dry Albumen) *
Total protein	-	100	9.7–10.6
Ovomucoid	Gal d 1	11	1.17
Ovalbumin	Gal d 2	54	5.72
Ovotransferrin	Gal d 3	12	1.27
Lysozyme	Gal d 4	3.5	0.37

^ Codes are established by the IUIS Allergen Nomenclature Sub-Committee; * data for single protein calculated on 10.6% of total protein.

#### 3.1.2. Methods for Quantification of Residual Egg Proteins

The ELISA test is the most important analytical method applied to the detection of allergens in foods, because of its specificity and sensitivity, and since the equipment required is not expensive. According to the new regulation, wine labeling exemption can be maintained only if: (1) egg white proteins are not used and cross-contamination is under control; (2) wine clarified with such products tested negative for the presence of residues using techniques with a detection and quantification limits of 0.25 mg/L and 0.5 mg/L, respectively. Analytical requirements were defined by the International Organization of Vine and Wine (OIV) by resolution 427-2010 [13], modified by OIV/COMEX 502-2012 [14]. Several ELISA tests have been developed internationally in accordance with the OIV rules [15,16,17,18,19,20]. Some authors have developed alternative methods based on different analytical approaches such as mass spectrometry [21,22]. These methods are highly sensitive but are not suitable for routine monitoring of egg proteins in wines because they are not traceable to the threshold value.

#### 3.1.3. International Studies on Wines Fined with Egg Derivatives

Several studies investigating the presence of residual egg white proteins in fined wines are listed in Table 2. In this context, Weber *et al.* [19] measured by an ELISA the presence of allergenic residues in four experimentally developed wines fined with egg white proteins and treated or not treated with bentonite. The authors showed no detectable amounts of egg white proteins in wines fined with the dose recommended by the manufacturer (4 g/hL of wine). Egg white proteins were only detected at a level of 0.2 mg/L in one wine fined with a dosage of egg white (20 g/hL) five times higher than the recommended one. Since the thresholds for allergic reactions to egg white are approximately 1 mg (as a single dose), the authors concluded that the consumption of approximately 1.2 L of wine treated with dried egg white would be necessary to trigger an allergic reaction in sensitive individuals. Results obtained by Weber *et al.* emphasized the need for further investigation in a larger number of commercial wines. In order to fill this gap, Rolland *et al.* [18] applied an ELISA assay to detect ovalbumin in 40 commercially available Australian wines fined with egg white proteins. None of the studied wines showed ovalbumin traces at the level of detection of the method (limit of detection, LOD: 1 mg/L). In another study performed in three experimental red wines and 400 commercially available bottled wines fined with albumin and albumin with lysozyme, Lifrani *et al.* [16] revealed by a sandwich-ELISA the presence of traces of egg white after wine filtration. The wines that were positive in the ELISA were given to mice sensitized to egg white in order to verify their allergenicity. No anaphylactic reactions were detected in the animals. Recently, an ELISA assay was specifically developed to detect traces of egg white proteins in wine [23]. The presence of allergenic egg white residues in 14 experimental and 78 commercially available wines was evaluated using the newly developed ELISA [9]. None of the analyzed wines contained detectable amounts of egg white proteins, irrespectively of the fining agent dose and the enological practice used. The absence of egg white proteins in these wines was confirmed by immunoblotting (with similar limit of detection). Similarly, Deckwart *et al.* [15] studied by two different ELISA assays whether egg proteins were completely removed in wines fined with egg white and treated by different technological procedures to remove the fining agents. The authors observed that wine can retain egg white proteins when not treated by good manufacturing practices. After the use of bentonite or sheet filtration followed by sterile filtration egg allergens in wine were not detectable. Deckwart *et al.* [15] also examined whether the analyzed wines could elicit allergic reactions in five sensitized subjects. None of the individuals reacted to fined wines in the skin prick test. The oral provocation was performed with wines containing ovalbumin below the limit of detection of the ELISA assay. All individuals tolerated the maximal dose of 200 mL of fined wine without any clinical reaction.

Recently, a few studies have applied mass-spectrometry (MS) to detect residues of fining egg proteins in wines (Table 2). For instance, Tolin *et al.* [24] analyzed a panel of 25 commercial Italian wines treated with doses of egg white as low as 5 g/hL by liquid chromatography coupled with tandem MS (LC-MS/MS) in a gel-free approach in order to detect the occurrence of traces of egg white proteins [25]. They found ovalbumin in eight of the wines studied, while the allergen ovotransferrin was identified in two of them. On the basis of the limit of detection of the method, the minimal amount of egg white proteins in the studied wines was estimated to be 100 ng/L. Similarly, Mattarozzi *et al.* [26] proposed an LC-MS/MS method for the simultaneous determination of residues of ovalbumin and milk caseins in red wine. A total of 20 Italian commercial wines were analyzed by the described method, and no traces of peptides from ovalbumin were found in the samples (LOD: 0.8 μg/mL).

**Table 2:** Studies reporting data on allergenic residues in wines fined with egg white.

Type of Wines	Number of Wines	Fining Agent	Dose (g/hL)	Analytical Method	Results of the Study	Reference
EW	4	Egg white	4, 20	ELISA	Detection of egg white proteins (0.2 mg/L) wines fined with 20 g/hL; no detectable amounts of egg white proteins in wines fined with 4 g/hL	[ 19]
CW	24	Egg white Whole egg	NS	DBPCFC Blood basophil activation	Lack of anaphylaxis and basophil activation	[ 27]
CW	40	Egg white	5–15	ELISA	No detectable amount of egg white proteins	[ 18]
EW CW	3 400	Egg white proteins (with/without lysozyme) NS	3, 10 3	ELISA Challenge in mice sensitized to albumin	Detection of egg albumin in some wines Lack of anaphylaxis with positive wines in sensitized mice	[ 16]
CW	5	Egg white proteins	20	DBPCFC SPT	Lack of anaphylaxis No skin reaction	[ 28]
CW	25	NS	NS	LC-MS/MS	Detection of ovalbumin (8 wines) and ovotransferrin (2 wines)	[ 25]
EW (different treatments)	2	AlbuVin™	16	ELISA DBPCFC	No detectable amounts of egg white in wines produced with good enological practices Lack of anaphylaxis	[ 15]
EW CW	14 78	Egg white proteins Commercial egg white products	3–10 1–66	ELISA Immunoblotting	No detectable amounts of egg white proteins	[ 9]
CW	20	NS	NS	LC-MS/MS	No detectable amounts of egg white proteins	[ 26]
CW	8	NS	NS	ELISA	7 wines below LoD, one at 1.7 mg/L of whole egg powder concentration	[ 20]

EW = experimental wine; CW = Commercial wine; ELISA: enzyme-linked immunosorbent assay; DBPCFC: double-blind, placebo controlled food challenge. LC-MS/MS: liquid chromatography coupled with tandem mass spectrometry; NS: not specified; SPT: skin prick test.

Two studies involving individuals allergic to egg white have been reported in the last decade (Table 2). In the first one, Rolland *et al.* [27] performed a double-blind, placebo-controlled food challenge (DBPCFC) trial to determine whether the consumption of Australian commercial wines fined with egg white proteins could induce anaphylaxis in a group of five adults allergic to egg. Twenty-four white and red wines fined with egg white or whole egg and two control red wines without these fining agents were included in the research. No significant clinical response attributable to the consumption of fined wines was observed in any of the subjects participating in the study. In 2009, Kirschner *et al.* [28] carried out another DBPCFC aimed to investigate whether traces of egg white proteins used as processing aid during wine manufacturing could trigger an allergic reaction in individuals susceptible to egg. The study included German white wines fined with egg albumin at a dose five times higher than that used in commercially available wines and also unfined wines (control wines). Five adults allergic to egg were recruited for the study. Despite the allergenic potential showed by ovalbumin fining agent in skin prick test, no skin reactivity to fined wines was observed. Moreover, none of the allergic individuals showed an adverse reaction in the oral wine provocation test with the consumption of 200–300 mL of wine. These results suggest that the risk of allergic reactions followed the consumption of wines fined with egg white proteins is negligible. However, these studies included only five individuals allergic to egg, and therefore the statistical reliability of the study is low. Further studies using a larger panel of allergic individuals are necessary to confirm the lack of allergenic reactions elicited by traces of fining agents contained in wines.

#### 3.1.4. Assessment of Possible Risk for Allergic Subjects

The EFSA Panel on Dietetic Products, Nutrition and Allergies (NDA) was asked by the International Organization of Vine and Wine (OIV) to deliver a scientific opinion related to the possibility to receive a permanent exemption from labeling of ovalbumin/egg white proteins used as fining agents [29]. The Panel concluded that a generalized exemption could not be made because of the lack of standardization of the wine manufacturing process and the absence of sufficient clinical data showing tolerance of treated wines by sensitized subjects. Unfortunately, the production of data in line with EFSA requirements is quite difficult since wines are produced with significantly different enological protocols and the number of adult subjects allergic to egg is so small that statistical significance cannot be reached.

### 3.2. Milk Proteins

#### 3.2.1. Enological Applications

Milk, skim milk and more frequently caseinates are used as fining agents. The latter are usually added to white wines in form of potassium caseinate, which is more soluble than casein itself [17]. Caseinates improve filterability and are efficient in eliminating the oxidative browning products [16], that is an important defect in white wines. Flocculation due to the precipitation of caseinates at acidic pH is rapid, and removal is facilitated by the addition of secondary fining agents such as bentonite [30]. The amount of caseinates usually added ranges between 5 and 50 g/hL (50–500 mg/L), and no risk of over-fining is likely due to the low solubility at low pH values. Like egg proteins, caseinates have useful enological properties, but any residues left in the fined wines could constitute a risk for the rare individuals suffering from milk allergy in adulthood [17]. Milk contains several allergenic proteins listed in Table 3 [31], but only caseinates are customarily added to wines.

**Table 3:** Main allergens of cow’s milk.

Protein	International Allergen Code ^	Protein Content (% Total Protein)	Protein Content (g/100 g Dry Milk) *
Total protein	-	100	26.15 *
Alpha-lactalbumin	Bos d 4	5	1.31
Beta-lactoglobulin	Bos d 5	10	2.62
Serum albumin	Bos d 6	1	0.26
Immunoglobulin	Bos d 7	3	0.78
Total casein	Bos d 8	80	20.92
AlphaS1-casein	Bos d 9	29	7.58
AlphaS2-casein	Bos d 10	8	2.09
Beta-casein	Bos d 11	27	7.06
Kappa-casein	Bos d 12	10	2.62

^ Codes are established by the IUIS Allergen Nomenclature Sub-Committee (www.allergen.org); * 13% total solid and 3.4 g/L of proteins.

#### 3.2.2. Methods for Quantification of Residual Milk Proteins

As reported for egg proteins, the ELISA test is the most usual analytical method applied to detect caseinates or milk proteins in wine. The same OIV guidelines for analytical methods described in Section 3.1.2., including LOD/LOQ, are applied to milk proteins [13,14]. Several scientific papers have illustrated methods developed to monitor wines fined with milk proteins; most of them are ELISA methods in agreement with the OIV guidelines [16,17,18,19,23,32,33,34], while other authors have used mass spectrometry [10,21,22,35].

#### 3.2.3. International Studies on Wines Fined with Milk Proteins

Various *in vitro* studies based on immunological methods have been conducted to evaluate whether traces of milk proteins used for fining processes can remain in the finished wine (Table 4). Weber *et al.* [19] applying an ELISA assay with LOD of 0.10 mg/L showed no detectable amounts of caseins in four experimental wines fined with 6 and 30 g/hL of potassium caseinate. In another study, the same research group studied experimental and commercial wines using western blot with immunostaining and indirect ELISA assays [33]; they found caseins (0.2–0.4 mg/L) in five out of 93 wines tested. They also showed that treatment with bentonite followed by cross-flow filtration left no detectable casein residues. Rolland *et al.* [18] analyzed a panel of 153 commercially available Australian wines by ELISA and concluded that they were free from detectable casein residues. These findings agree with those reported by Restani *et al.* [17], who evaluated 79 experimental and commercial wines fined with caseinates and observed no detectable allergenic residues in any of the samples using immunochemical and ELISA assays. Results were negative irrespective of the physicochemical wine characteristics, the enological process used and the type and dosage of the fining agent. In contrast, Lifrani *et al.* [16] studied a panel of more than 400 wines both prepared in the laboratory and commercially available, and found caseinate in both types of wines also using an ELISA assay. The wines with positive ELISA results were injected into mice sensitized to caseinate and no anaphylactic reaction was observed. In a recent study, Deckwart *et al.* [32] observed by ELISA (LOD: 0.1 mg/L) negligible residual allergenic protein in experimental red and white wines fined with casein from different commercial sources.

Recently, several studies based on the detection of milk fining agents in wines by mass spectrometry methods have been reported (Table 4). Monaci *et al.* [36] used capillary liquid chromatography combined with electrospray ionization-tandem mass spectrometry (CapLC-ESI-MS/MS) and found residual α and β caseins in experimental white wines fined with caseinate. Tolin *et al.* [25] were also able to detect traces of caseins in two of 25 commercial wines by LC-MS/MS.

The risk of anaphylactic reactions in milk allergic subjects triggered by traces of caseins remaining in wines after fining has been evaluated by two DBPCFC trials (Table 4). Rolland *et al.* [27] conducted a trial to investigate whether a panel of 59 wines fined with casein elicited an allergic reaction in individuals allergic to milk. This study suggested that wines produced according to good manufacturing practice present a negligible risk of inducing a clinically significant adverse reaction in adult subjects with confirmed allergy to milk. However it is worth noting that only one milk-allergic subject was included in the study since milk allergy is very rare in adults, so that no statistically relevant conclusions can be obtained from this study. The results reported by Rolland *et al.* were supported by those obtained by Kirschner *et al.* [28], who performed a DBPCFC in five milk-allergic individuals with five experimental wines fined with potassium caseinate at a dose five times higher than that usually applied in commercial wines and did not observed any clinical reaction after consumption of 200–300 mL of fined wines.

#### 3.2.4. Assessment of Possible Risk for Allergic Subjects

The EFSA Panel on Dietetic Products, Nutrition and Allergies (NDA) was asked by the International Organization of Vine and Wine (OIV) to deliver a scientific opinion related to the possibility to receive a permanent exemption from labeling of milk derivatives used as fining agents [37]. As for egg derivatives, the Panel concluded that a general exemption could not be made because to the lack of standardization of the wine manufacturing process and the absence of sufficient clinical data showing tolerance of treated wines by sensitized subjects.

### 3.3. Isinglass/Fish Gelatine

#### 3.3.1. Enological Applications

Isinglass is a positively charged fining agent derived from the air bladder of certain fish species such as sturgeon. Isinglass is used in still, dry, sweet and sparkling white wines to clean up the aroma, improve clarity and modify the finish without significantly modifying tannin levels. The flocculation is fast due to the low solubility of isinglass at wine pH, while the sedimentation is normally facilitated by the use of a secondary fining agent, such as bentonite. Usage levels are typically from 2.5 to 6 g/hL (equivalent to 25–60 mg/L) [8]. The main proteins of isinglass and their allergenic potential are reported in Table 5.

**Table 4:** Studies reporting data on allergenic residues in wines fined with milk derivatives.

Type of Wines	Number of Wines	Fining Agent	Dose (g/hL)	Analytical Method	Results of the Study	Reference
EW	4	Potassium caseinate	6, 30	ELISA	No detectable amounts of caseins	[ 19]
CW	34	Casein	NS	DBPCFC Blood basophil activation	Lack of anaphylaxis and basophil activation	[ 27]
CW	153	Casein Skim milk	10–50 0.5%	ELISA	No detectable amounts of caseins	[ 18]
EW CW	32 61	Potassium caseinate	6, 30 with/without bentonite	SDS-PAGE/Immunoblotting ELISA	Detection of caseins in 5 out of 93 wines	[ 33]
EW	5	Potassium caseinate	30	DBPCFC SPT	Lack of anaphylaxis No skin reaction	[ 28]
EW CW	4 400	Caseinate NS	10–60 NS	Sandwich-ELISA Challenge in sensitized mice	Detection of casein in experimental and commercial wines Lack of anaphylaxis in sensitized mice	[ 16]
EW	2	Caseinate	10, 100	CapLC-ESI-MS/MS	Detection of residual caseins	[ 36]
EW CW	16 63	Caseinate	20, 50 2–55	Immunoblotting ELISA	No detectable amount of caseins	[ 17]
EW (different treatments)	NS	KalCasin	40, 80	ELISA	No detectable amount of caseins	[ 32]
CW	25	NS	NS	LC-MS/MS	Detection of caseins in 2 out of 25 wines	[ 25]
CW	20	NS	NS	LC-MS/MS	No detectable amounts of caseins	[ 26]

EW = experimental wines; CW = Commercial wines; CapLC-ESI-MS/MS = Capillary liquid chromatography combined with electrospray ionization-tandem mass spectrometry; ELISA: enzyme-linked immunosorbent assay; DBPCFC: double-blind, placebo controlled food challenge; LC-MS/MS: liquid chromatography coupled with tandem mass spectrometry; NS: not specified; SPT: skin prick test.

**Table 5:** Main proteins from isinglass and their allergenic potential.

Protein	Allergenic Potential	Protein Content ^
Parvalbumin	high	0.2–0.7 mg /kg isinglass
Type I + collagen and gelatine as collagen denaturation product	low	95% dry weight
Elastine	low	2.5% dry weight

^ Data from EFSA [38].

#### 3.3.2. Methods for Quantification of Residual Isinglass/Fish Gelatine

Because of the permanent exemption from labeling of isinglass in Directive 2007/68/EC [2], the development of analytical methods has been less active. The Brewing Food & Beverages Industry Suppliers Association (BFBi) and the British Beer and Pub Association (BBPA) commissioned two groups of independent experts in food allergies and brewing science to measure the residues of parvalbumin using an anti-cod parvalbumin polyclonal antibody sandwich ELISA kit having an LOD of 0.20 mg/kg [38]. A further indirect ELISA method was developed by Weber *et al.* in 2010 [39].

#### 3.3.3. International Studies on Wines Fined with Isinglass/Fish Gelatine

Although fish are a well-known cause of food allergy, not many studies have evaluate the presence of traces of fish gelatin and isinglass used as proteinaceous fining agents in wine (Table 6). In 2007, Weber *et al.* [19] performed a study in four experimental wines to examine by competitive ELISA whether residues of fish gelatine and isinglass remain after fining and filtration. They found no detectable amounts of either proteinaceous fining agent in the tested wines. The same research group evaluated experimental and commercial wines fined with isinglass at different dosages (10 and 50 g/hL) and concluded that fined wines also treated with bentonite were free from isinglass residues [39]. In contrast, Lifrani *et al.* [16] examined more than 400 experimental and commercial wines fined with isinglass by ELISA and reported the presence of residual amounts of this fining agent in all experimental wines and in fewer than 10% of commercial wines. However, when wines with positive results in the ELISA assay were injected into mice sensitized to isinglass, no anaphylactic reaction was noted.

The risk of wine treated with fish proteins for allergic individuals was evaluated in two DBPCFC. The one performed in 2006 by Rolland *et al.* [27], enrolled 10 subjects allergic to fish and considered a panel of 23 commercial Australian wines. The study showed a lack of basophil activation and no clinical response attributable to consumption of wines fined with isinglass. Similarly, Kirschner *et al.* [28] reported that wines fined with isinglass and fish gelatin at a dose five times higher than that used in commercially available wines did not elicit any skin and anaphylactic reaction in a DBPCFC performed in four adults allergic to fish.

All *in vitro* and *in vivo* studies demonstrated that wines fined with fish gelatine and isinglass, even at doses higher than those commercially applied, presented a very low risk of an allergic response in consumers allergic to fish.

**Table 6:** Studies reporting data on allergenic residues in wines fined with isinglass/fish gelatin.

Type of Wines	Number of Wines	Fining Agent	Dose (g/hL)	Analytical Method	Results of the Study	Reference
EW	4	Isinglass Fish gelatine	50, 250 mL 10 and 50	ELISA	No detectable amounts of fish gelatine/isinglass	[ 19]
CW	23	Isinglass	NS	DBPCFC Blood basophil activation	Lack of anaphylaxis and basophil activation	[ 27]
EW	5	Isinglass Fish gelatine	250 mL 50 g	DBPCFC SPT	Lack of anaphylaxis No skin reaction	[ 28]
EW CW	4 400	Isinglass NS	20–25 NS	ELISA Challenge in mice sensitized to isinglass	Detection of isinglass in EW and CW Lack of anaphylaxis in sensitized mice	[ 16]
EW CW	8 60	Isinglass NS	50, 250 mL ^ NS	SDS-PAGE, Immunoblotting ELISA	Detection of isinglass only in EW not treated with bentonite; No detectable amounts of isinglass in CW	[ 39]

EW = experimental wines; CW = Commercial wines; ELISA: enzyme-linked immunosorbent assay; DBPCFC: double-blind, placebo controlled food challenge; NS: not specified; SPT: skin prick test. ^ With/without bentonite.

#### 3.3.4. Assessment of Possible Risk for Allergic Subjects

In 2007, the EFSA Panel on Dietetic Products, Nutrition and Allergies (NDA) was asked by Winemakers Federation of Australia (WFA) and the Australian Wine Research Institute (AWRI) to deliver a scientific opinion whether there could be a permanent exemption from labeling of wine fined with fish gelatine (isinglass) [40,41]. The Panel concluded that a general exemption could not be made since “*data submitted do not allow the Panel to assess the likelihood that isinglass used as fining agent in wine will trigger an allergic adverse reaction in susceptible individuals under the conditions of use stated by the applicant*”.

In the same year, the EFSA Panel on NDA received a similar request by the Brewing, Food and Beverage Industry Suppliers Association for exemption from labeling of beer fined with isinglass [38]. The dossier presented data on parvalbumin (the most important fish allergen) level in final beer, which was approximatively 5 ng/L. The safety of the negligible residue of isinglass in beer was confirmed by DBPCFC studies, where none of 21 fish allergic patients experienced any adverse effect after the intake of isinglass used in enology. The Panel conclusion was: “*On the basis of the data provided, the Panel considers that it is not very likely that isinglass used as clarifying agent in beer will trigger a severe allergic reaction in susceptible individuals under the conditions of production and use specified by the applicant*”. As a consequence of this positive opinion, the European Union established with the Commission Directive 2007/68/EC [2] the permanent exception for labeling at point 4 of the Annex IIIa: Fish gelatine or Isinglass used as fining agent both in beer and wine. 

### 3.4. Proteins from Vegetable Source (Wheat, Lupine, Pea and Potato Proteins)

At the beginning of this century, vegetable proteins were proposed as new fining agents as an alternative to animal gelatines when concerns raised over cases of Bovine Spongiform Encephalopathy (BSE) appeared. Some different vegetable derivatives have been considered: gluten (as such or hydrolyzed), lupine, pea [42,43] and more recently potato proteins. Peas and potatoes are not included in the list of main allergens so that their inclusion in labeling is not mandatory. The experimental trials performed with lupine proteins, as a fining agent, showed that no residue was detectable in the final red wines, while a number of white wines presented ambiguous results [43]. Secondary treatment with bentonites was sufficient to remove the overall immunoreactivity but considering the good performance of pea proteins, no approval was required for the use of lupine proteins in enology. Consequently, only gluten (wheat) proteins will be considered here.

#### 3.4.1. Enological Applications

As described before, the incidence of BSE led to concerns about the use of proteins derived from animal sources in winemaking. Amongst the alternatives researched, wheat gluten as such [44,45] or hydrolyzed [44], were suggested as clarifying agents. Attention was paid mainly to hydrolyzed gluten, which is added to must or wine at doses ranging between 2 and 30 g/hL. This substance binds colloids and tannins with precipitation of the complexes formed; the wine is normally filtered to remove the possible residues of fining agents.

#### 3.4.2. Methods for Quantification of Gluten Derivatives in Wine

Because of their limited use in enology, few methods have been developed to detect residues of gluten derivatives in wines and musts. OIV included in the compendium of official methods the determination of plant proteins in wines and musts by an immunoelectrophoretic approach (electrophoretic separation and immunochemical detection) [46].

#### 3.4.3. International Studies on Wines Added with Gluten Derivatives

Only three papers have been published on the presence of immunoreactive residues in wine treated with gluten derivatives [44,47,48]. Two pathologies are associated with gluten, wheat allergy and celiac disease, which have different pathogeneses. Cattaneo *et al.* [44] looked for the presence of residues in seven red wines and 10 white wines where gluten derivatives (as such or hydrolyzed) were added to must (4) or wine (6) at a level of 10–50 g/hL. White wines were also fined in parallel with a secondary agent such as bentonite, silica gel or tannin. The residues where sought by the OIV official method with an LOD of 0.36 mg/L. No red wine showed any detectable residue in the final product. By contrast, some white wines presented traces of gluten independently of the use and type of secondary fining agent; among the secondary agents, bentonite was the most effective in removing gluten residues. Some immunoreactivity was detected by immunoblotting using animal anti-gliadin antibodies and IgAs from celiac patients, but none with IgEs from allergic subjects. Simonato *et al.* [48] measured by immunochemical assay and mass spectrometry the residues of gluten in red wines fined with partially hydrolyzed and non-hydrolyzed wheat gluten. Immunochemical methods (using anti-prolamin antibodies, anti-gliadin antibodies and sera-IgEs) detected residual antigenicity in wines fined with partially hydrolyzed gluten at concentrations 50–300 g/hL, whereas no residual proteins were detected by these systems in the wine treated with nonhydrolysed product. In contrast liquid chromatography-mass spectrometry allowed the detection of proteins in red wines fined with both products down to 1 g/hL.

#### 3.4.4. Assessment of Possible Risk for Allergic and Celiac Subjects

In 2004, the EFSA Panel on Dietetic Products, Nutrition and Allergies (NDA) was asked by SOFRALAB for permanent exemption from labeling of wines treated with hydrolyzed wheat gluten [49]. According to the scientific dossier presented, the EFSA Panel concluded that: “*The scientific data provided by the applicant are insufficient to predict the likelihood of adverse reaction in cereal allergic individuals. Nevertheless, taking into account the level of wheat proteins reported to cause allergic reactions in severely allergic individuals, the Panel considers that wines and musts treated with hydrolyzed wheat gluten could trigger an allergic reaction*”. More positive was the opinion for celiac subjects, since the Panel stated “*For coeliac disease, assessment of the provided evidence indicates that wines and musts treated with hydrolyzed wheat gluten are unlikely to cause an adverse reaction....*”*.*

## 4. Use of Allergenic Proteins as Additives

At present, the only allergenic protein used as an additive in winemaking is the enzyme lysozyme. As described at Section 3.1.1., lysozyme is the allergen Gal d 4 from egg white.

### 4.1. Lysozyme

#### 4.1.1. Enological Applications

Lysozyme, used as an antimicrobial agent, has very interesting activity in enology since, having a strong inhibitory effect on Gram-positive bacteria, it is a natural and efficient alternative to reduce the use of sulfites. Lysozyme is included among wine additives able to control fermentation processes and avoid spoiling during winemaking [50].

Lysozyme is generally considered as a safe product, which has been positively evaluated by several regulatory agencies and which has a safe track record of use during many years in the pharmaceutical and food industries.

Being an egg allergen (see Section 3.1.1.), the critical point is again the safety of wines treated with lysozyme for subjects suffering from food allergy, since the additive remains in final wine, if it is not removed by a secondary specific treatment. 

The amount of lysozyme added normally ranges between 25 and 50 g/hL (250–500 mg/L). Four main applications and dosages are: (a) prevention of the onset of malo-lactic fermentation (early addition of 10–15 g/hL); (b) total inhibition of malo-lactic fermentation (50 g/hL); (c) protection of wine during sub-optimal alcoholic fermentation (25–30 g/hL); (d) stabilization of wine after malo-lactic fermentation (25–30 g/hL). The lysozyme is eliminated by the secondary addition of fining agents; among which bentonite and metatartaric acid are the most efficient.

#### 4.1.2. Methods for Quantification of Lysozyme

Some analytical methods have been developed to check for residues of lysozyme before the treated wines are bottled. Various approaches are used: ELISA [19,51,52]; high performance liquid chromatography (HPLC) [53,54]; mass spectrometry [21,55], and capillary electrophoresis [56]. Other methods, e.g., immunoblotting and a microbiological test, have been used in experimental studies [57].

#### 4.1.3. International Studies on Wines to Which Lysozyme Has been Added

One study [19] measured the residues of lysozyme in four German wines experimentally produced with lysozyme and clarified with bentonite. The values measured by ELISA kit ranged between 0.01 and 0.06 mg/L. Further HPLC analysis performed by the same researchers [51], showed that wines treated with lysozyme and not fined with bentonite could contain residues up to 327 mg/L in white wines, and up to 38 mg/L in red wines (Table 7). Since the use of bentonite is not mandatory in winemaking, the authors concluded that allergic reactions to lysozyme-treated wines could be possible after moderate wine consumption (0.1–0.7 L) by the most susceptible individuals. Lysozyme residues were also determined in 29 commercial lysozyme-treated wines (three white, two rosé, and 24 red wines) produced in six countries (Argentina, Australia, France, Italy, Spain, USA) [57]. Wines were treated with 3–50 g/hL of lysozyme with or without secondary fining with metatartaric acid and/or bentonite. Most of them were filtered (porosity between 0.45 and 5.3 μm). Two wines, analyzed by immunoblotting using an anti-lysozyme antibody, contained lysozyme residues of 8.6 and 2.6 mg/L. The presence of residues was confirmed by HPLC and by a microbiological method.

**Table 7:** Studies reporting data on allergenic residues in wines to which lysozyme has been added.

Type of Wines	Number of Wines	LYS and Secondary Treatment	Doses (g/hL)	Analytical Method	Results of the Study	Reference
EW	4	LYS + bentonite	25 and 50	ELISA	Residues were 0.001–0.06 mg/L	[ 19]
EW	5	LYS + bentonite	25, 50 0–700	HPLC	Without bentonite fining, the residues of LYS detected in final wines were significantly higher. In white wines, LYS concentration was ≤183 mg/L and 327 mg/L, for addition of 250 and 500 mg/L, respectively. In red wines, ≤27 mg/L and 38 mg/L, with 250 mg/L and 500 mg/L of LYS added, respectively	[ 51]
CW	29	LYS + Bentonite/metatartaric acid	5–50	Electrophoresis/Immunoblotting HPLC Biologic test	Two positive wines with LYS residues of 8.6 mg/L and 2.6 mg/L, respectively; three other wines scored positive at immunoblotting after concentration	[ 56]

EW = experimental wines; CW = Commercial wines; ELISA: enzyme-linked immunosorbent assay; HPLC: high-performance liquid chromatography; LYS: lysozyme.

#### 4.1.4. Assessment of Possible Risk for Allergic Subjects 

Lysozyme is a minor allergen among egg white proteins, and the most severe sensitization is normally due to professional exposure by inhalation. In this case, the subjects sensitized to lysozyme tolerated this protein taken orally, as shown by DBPCFC in two subjects having anaphylaxis at inhalation but no reaction to cheese or wine treated with lysozyme (Restani, unpublished data). However, it is difficult to determine the true prevalence of food allergy involving lysozyme. In 2011, the EFSA Panel on Dietetic Products, Nutrition and Allergies (NDA) was asked by OENOPPIA (Oenological Products and Practices International Association) for permanent exemption from labeling of wines treated with lysozyme [58]. According to the scientific dossier presented and the clinical data at its disposal (see also Section 4.1.3), the EFSA Panel concluded that: “*Wines treated with lysozyme may trigger adverse allergic reactions in susceptible individuals under the conditions of use proposed*”.

## 5. Conclusions

The use of organic substances in the clarification of wines is quite common; among them, animal proteins (egg, milk and isinglass) are the most widely used for their excellent fining properties and their relative favorable prices. More recently products from vegetable source have been proposed as an alternative to animal derivatives: gluten, lupine, pea and potato proteins. With the most recent European legislation relative to allergenic ingredients, wine producers had to face the problem of labeling, as a consequence of the extension of the rules to alcoholic beverages.

Because of the negative reactions of consumers at the indication of animal proteins on a wine label, several studies have been performed to evaluate the possibility to receive permanent exemption from labeling in Europe. Several opinions were prepared by EFSA in relation to this topic, which were usually negative (only isinglass received permanent exemption based on a positive opinion by EFSA concerning its use in beer). The main conclusions from all information given in this review can be summarized as follows:

(1) All studies measuring the residues of allergenic additives/fining agents showed that most wines at bottling were free from allergenic proteins, but in some cases relatively high quantities of them can be still present (egg white proteins more frequently than milk proteins);
(2) Although allergy to milk and egg is quite rare in adults, some subjects can present severe clinical reactions, including anaphylactic shock;
(3) The removal of allergenic additives or fining agents can be optimized by specific guidelines, which include filtration and, in some cases, a secondary fining treatment (see OIV guidelines) [59];
(4) To avoid labeling of wine fined with allergenic proteins, rigorous checking for residues is recommended. Among the recognized methods of analysis, an ELISA test seems the most suitable for sensitivity, specificity and its ease of use by laboratories routinely dealing with wine or food analyses. All available commercial kits, which must be specifically validated for wine, have a Limit of Detection and Limit of Quantification similar to or lower than values established by OIV, namely 0.25 and 0.5 mg/L, respectively (OIV 2010, modified 2012);
(5) The OIV guidelines can be quite easily followed by winemakers, and they can therefore avoid labeling without risk for allergic subjects.
